# Non-Suicidal Self-Injury: A School-Based Peer Education Program for Adolescents During COVID-19 Pandemic

**DOI:** 10.3389/fpsyt.2021.737544

**Published:** 2022-01-13

**Authors:** Annarosa Cipriano, Cristina Aprea, Ludovica Bellone, Paolo Cotrufo, Stefania Cella

**Affiliations:** Observatory on Eating Disorders, Department of Psychology, University of Campania “Luigi Vanvitelli”, Caserta, Italy

**Keywords:** non-suicidal self-injury, peer-education, prevention, adolescence, puberty

## Abstract

**Introduction:** Non-suicidal self-injury (NSSI) constitutes a major health concern among youth. However, less is known about the useful ways to prevent NSSI. As such, the NSSI- Peer Education Program (NSSI-PEP) aims to intervene on the vulnerability factors that predispose to NSSI by applying a peer education approach. The NSSI-PEP is grounded on the psychoanalytic tradition's tenets, implementing modules targeting four crucial risk factors for NSSI: pubertal transformation, body image, self-esteem, and emotion regulation.

**Methods:** Selected 8^th^ grade students were trained to serve as peer educators and held a peer-education intervention for 6^th^ and 7^th^ grade students. Pre- and post-intervention assessments were conducted in order to evaluate the program's effectiveness.

**Results:** Results revealed preliminary support for the program's feasibility, as students reported greater emotion regulation abilities (*p* = 0.038) and significant changes in self-esteem (*p*<*0.0*01), personal alienation (*p* = 0.005), body image (*p* < 0.001), and maturity fear (*p* < 0.001). Also, NSSI-PEP was positively evaluated by participants.

**Discussion:** Our pilot study provides preliminary empirical support for the NSSI-PEP, representing a promising way to address areas of vulnerability for NSSI onset. Findings may also help current policies to promote targeted preventive activities and produce sizable benefits to society.

## Introduction

Non-suicidal self-injury (NSSI) is a clinical condition that refers to the socially unacceptable and intentional destruction of one's body tissue in the absence of suicidal intent (1). Common methods include cutting and severe scratching (2). Approximately 17% of adolescents report a lifetime history of NSSI (3), with 7.6% meet the criteria for the proposed NSSI disorder (4). Research has suggested that NSSI onset typically occurs during youth, increases across development from childhood through adolescence, peaking around the age of 14–15 years (5), and commonly ceases in young adulthood (6).

Although there is a paucity of research among youth under 12, findings suggest an increasing prevalence in early adolescence, with first episodes occurring at age 11 or younger (5). This is quite alarming given that those who engage in NSSI sooner (i.e., before 12 years) have a greater odd of engaging in a more severe pattern of NSSI [e.g., greater frequency, longer duration, and multiple methods; (7)]

Despite the lack of immediate lethality, NSSI constitutes a major public health problem as self-injurers are at higher risk for attempting suicide (8), and adverse mental health outcomes such as depression, anxiety, and eating disorders, as well as maladaptive behaviors, such as substance use (9).

Such adverse consequences are of particular interest given that the majority of youth who self-injure do not seek professional help (10) and are reluctant to self-disclosure due to the fear of negative reactions and stigmatization (11). Besides being highly disabling in its own right, NSSI also significantly impacts parents' wellbeing and parenting (12). After discovering their offspring's NSSI, parents report shock, fear, and guilt and experience both psychological/emotional and physical symptoms (13).

### Preventive Program for NSSI

Despite increased awareness that NSSI represents an impairing behavior impacting adolescents' functioning and taxing on parents, developing evidence-based prevention programs for youth is still limited. To the best of our knowledge, to date exist only two school-based prevention programs for NSSI: The Signs of Self-Injury [SOSI; (14)] and the HappylesPLUS (15). The SOSI program (14) provides psychoeducation to school staff/personnel and students. Significant changes in knowledge and attitudes toward NSSI, alongside the absence of iatrogenic effects (i.e., no increase in NSSI thoughts or behaviors), make SOSI a promising approach to NSSI prevention (16). However, although the program proves effect in improving NSSI understanding, it responses to the need of minimizing the negative effects once the behavior has occurred (tertiary prevention) rather than intervene to prevent the emergence of the behavior (primary prevention). Moreover, the pilot study neither evaluates variations in NSSI engagement nor changes regarding formal help-seeking actions of self-injurers. The HappylesPLUS (15) is a school-based prevention program for pupils (11–15 years) focusing on mental health that integrates a psychoeducational module on NSSI. Baetens et al. (15) found evidence that the program shows no iatrogenic effects, and students reported a reduced likelihood of future NSSI engagement.

Although both programs revealed that the NSSI rate was unchanged after module implementation, results are quite nascent and row, suggesting that more work needs to be done to develop an effective program for preventing NSSI. Aside from NSSI-specific programs, several prevention programs focus on youth mental health and suicide prevention, such as the Youth Aware of Mental Health program. That is a school-based universal intervention for adolescents implemented in the Saving and Empowering Young Lives in Europe (SEYLE) study (17) to raise mental health awareness about risk and protective factors associated with suicide, which has shown promising results. However, its efficacy for NSSI is unclear.

### Peer Education Methodology

The practices guidelines for prevention suggest that an optimal way of prevention program planning consists of identifying the factors contributing to the occurrence of the target behavior (18). As a result, over the past few decades, researchers have identified several factors that pose vulnerabilities to initiation and maintenance of NSSI during adolescence and support the need to integrate current NSSI's knowledge within preventive practice guidelines (19). Bearing such consideration in mind, asides from early NSSI onset, the NSSI-Peer Education Program (NSSI-PEP), presented in this study, proposes an innovative avenue of contacting youth, using a peer education (PE) approach in a secure setting (i.e., school) to address the psychological characteristics that have long been associated with NSSI among youth.

PE is an interactive approach indicating a “peer-to-peer education” relying on an informational exchange between credible, trusted peers and those being “educated” (20). During adolescence, youths are more sensitive to peers' influence (21). Thus, the PE approach assumes that peers, as experts by experience, are more credible than adults and have a non-judgmental horizontal position that makes it easier to contact and effectively influence the target group. As part of the same social group, peers have the desirable attributes of person-based (relating to demographic characteristics, such as age, sex), experience-based (based up sharing similar concerns and experiences), and message-based credibility [relating to non-moralistic communication and shared-language; (22)]. In this sense, peers are a positive source as they are able to provide support and foster change due to their shared affiliation and deep understanding of life experiences.

PE programs can be delivered in formal and informal settings, such as schools, clinics, and community centers (23). However, the school remains a privileged environment for delivering programs for youth and building protective competencies, as adolescents spend a greater part of their time at school (24).

Since the onset of NSSI is quite early, middle-school students may be the target population for a school-based prevention program based on the PE approach to avert the problem behavior and avoid potential pathways to suicide attempts.

### NSSI-PEP Program

#### Theoretical Framework

Adolescence is a rapid growth and development phase in physical and psychological domains (25), which also contributes to adolescents' increased risk for mental health problems, such as depression, anxiety, eating disorders, externalizing problems (26, 27), and adverse developmental outcomes (28).

In the psychoanalytic perspective, the developmental function of adolescence involves integrating the infant fantasies in the new muscular and sexual mature body–allowing now the acting-out–through several changes and adjustments (29). The traumatic potential of pubertal transformations, alongside the impulses' realizability and Oedipal conflict's re-actualisation, may disturb the adolescent's relationship with the body (30, 31), as adolescents may feel inadequate to facing the mounting excitement arising from the (sexual) body and trigger defense mechanisms. More specifically, feelings of abnormality and worthlessness, experienced by adolescents, are moved into the body with which adolescents seem to be in a war conflict (32). Thus, an interference in the integration of the sexual body image may lead adolescents to treat the body as an enemy (stranger), which could be disinvested and/or attacked (33). In this sense, adolescents are particularly vulnerable to attack their bodies, most aggressively, because they feel their pubertal bodies as responsible for their sensations, thoughts, and wishes, which are ultimately unbearable. Theoretical and empirical literature argues that the way one perceives and experiences the body influences the process of its emotional investments, which leads to a reduction in self-protection tendency and increases the likelihood to engage in self-destructive behaviors (34, 35).

The body is the central object in NSSI (34). However, although body disregard has long been associated with the occurrence of NSSI, research supports that it may also intervene in the development and maintenance of NSSI through various mechanism (36). Given that the body emotional investment is closely tied to self-esteem for adolescents and the demonstrated link with emotion regulation abilities (37–39), it is likely that both self-esteem and emotion regulation serve as mechanisms through which the body-image disruption operates.

Drawing from these assumptions, we have designed a theoretical-driven program–NSSI-PEP–to effect empirically selected psychological factors that contribute to the development of NSSI among youth, working on risk reduction and the enhancement of protective factors. The program aims to target the vulnerabilities factors that-both theoretically and empirically–have been associated with NSSI among youth: pubertal transformation, body image, self-esteem, and emotion regulation (40–42). Moreover, NSSI-PEP relies on implementing a peer-education approach representing a new way of working in this field to face NSSI.

#### Pubertal Changes and Body Image in NSSI

Rapid physical, endocrinological and psychosocial changes associated with puberty increase the relevance of body experienced during adolescence and leads youth to greater concerns about body image (43). Researchers demonstrated that a negative body image constitutes a critical risk factor for NSSI onset within clinical and non-clinical samples of adolescents (36, 42). Negative feelings and perceptions related to the body can make it easier to attack the body (35, 44) by encouraging feelings of devaluation and detachment from the body and reducing emotional investment in protecting physical integrity (34, 45). Further confirming such assumptions, current research demonstrates that individuals reporting a history of NSSI endorse significantly more body-related disturbances than those without a history of NSSI (46).

#### Self-Esteem and NSSI

As the body is the focal point of adolescence developmental processes, it is not surprising that adolescents struggle with self-esteem. Although general self-esteem is relatively stable, it becomes increasingly domain-specific over the life course, as individuals encounter new roles and situations (47) and life transitions, such as one attributable to pubertal changes, are salient in exerting a strong influence on how one judges own value.

Self-esteem has demonstrated a deep relationship with NSSI, as it tends to be deficient in self-injuring individuals who show lower scores than those not engaged in NSSI (48). Negative self-esteem has been found to be a salient predictor for NSSI (49, 50). Also, those who engage in NSSI show greater impairment in concepts aligned with self-esteem, such as self-criticism, ineffectiveness, and self-blame (51–53). Cumulatively, these findings demonstrate that self-esteem is inversely related to NSSI engagement and its frequency (54), suggesting that enhancing individual self-esteem may be a protective factor against NSSI (55).

#### Emotional Regulation and NSSI

Emotion dysregulation is a transdiagnostic factor common to many psychopathologies, such as binge eating (56), and has been identified as a core vulnerability for NSSI (57). A review on the topic has shown how people with NSSI exhibit a lower ability to recognize, understand, and express emotions (40), and difficulties in emotion regulation have been positively correlated with NSSI frequency, noting that a lower regulation can lead to a higher number of NSSI behaviors (58). In this sense, NSSI represents a (maladaptive) coping strategy to reduce/avoid intense negative feelings (59). Consistently, a large body of research suggests that those who repetitively harm themselves are more prone to use NSSI to reduce negative affects (60), while those who can regulate their aversive emotions are less likely to engage in NSSI (61).

#### The PEP-NSSI Objectives

The PEP-NSSI is an interactive, participatory, and empowering approach based on the assumption that intervening early on NSSI vulnerability factors could prevent youth from engaging in NSSI. Thus, the PEP-NSSI aims to: a) intervene early to reduce the risk for NSSI among youth while enhancing protective factors through improving emotion regulation abilities, feelings of self-esteem, and building a positive relationship with own body image; b) conduct an initial evaluation of the effectiveness and feasibility of the NSSI-PEP program; c) gather helpful suggestion for the development of evidence-based prevention programs and improve the effectiveness of social policies for the promotion of healthy behaviors for adolescents. Thus, we hypothesized that the program would enhance emotion regulation abilities, improve self-esteem, and develop a positive body image.

## Materials and Methods

### Study Design

**Figure 1:**
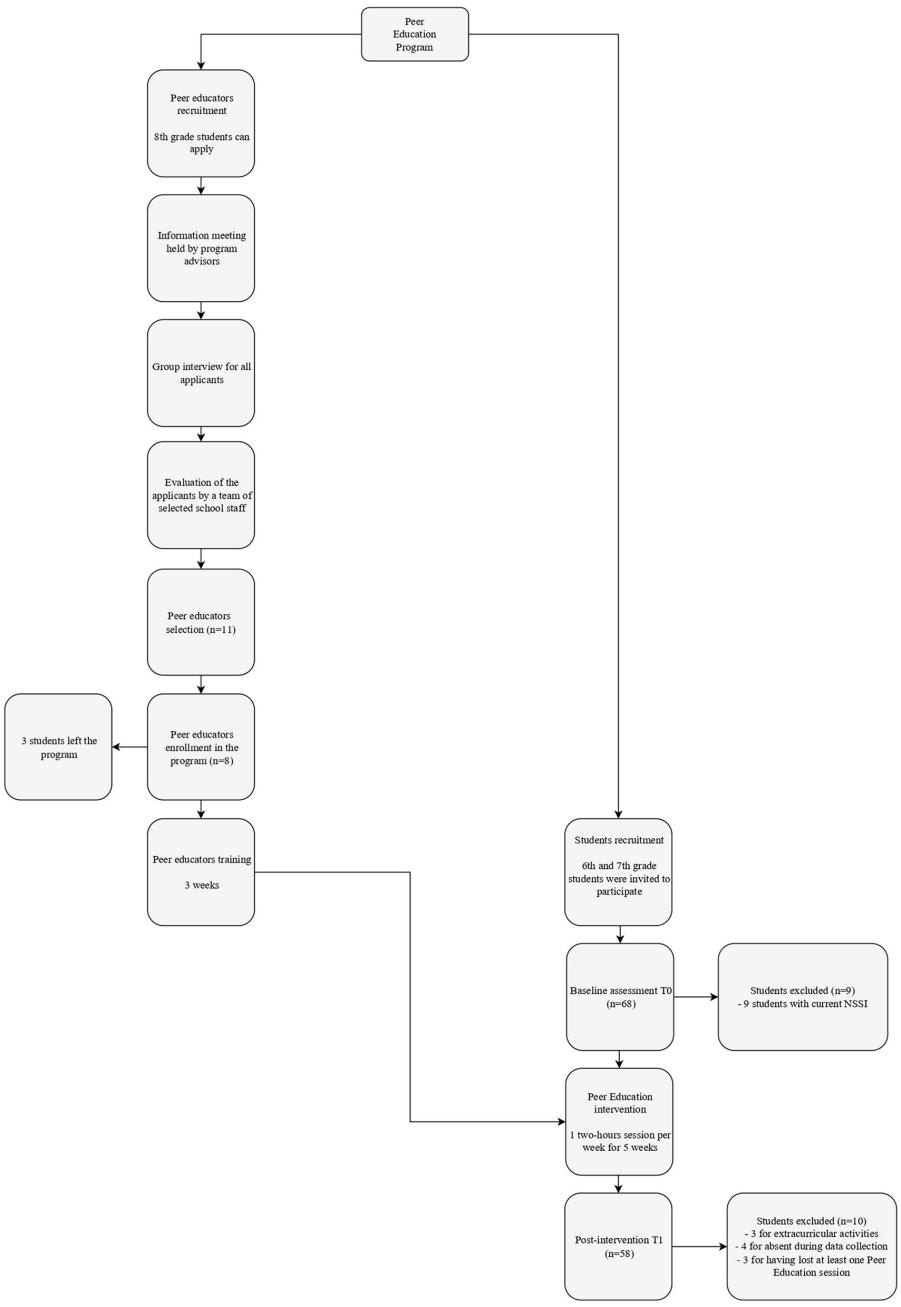
Flowchart of the peer education intervention.

The PEP-NSSI was a pilot uncontrolled feasibility study using a nonrandomized design and involved a middle school in Southern Italy. The program aimed to increase students' knowledge, skills, and behaviors and enhance psychological characteristics (i.e., self-esteem, body image linked to pubertal maturation, and emotion regulation) that pose vulnerabilities to NSSI among youth. See Figure 1 for the Flowchart of the intervention.

### Participants and Procedure

After the school headmaster agreed to participate, the NSSI-PEP was implemented in the school setting and integrated into the school day. Due to the restrictions introduced in response to the COVID-19 pandemic, all the activities (peers' formation and PEP intervention) were conducted online, using a digital platform (Google Meet). It occurred at two consequent stages. Peer educators were selected and trained to acquire the knowledge and skills required to lead PE to younger students in the first stage. In the second stage, research participants received a PE intervention over 5 weeks. Also, they were screened at baseline and after PEP completion. Participants were informed of the importance of attending each intervention's sessions.

All procedures were reviewed and approved by the Ethics Board of the Department of Psychology-University of Campania “Luigi Vanvitelli.” The study was conducted in accordance with the principles of the Declaration of Helsinki (62). The study protocol followed the International Conference on Harmonization (ICH) guidelines for E3 (Structure and Content of Clinical Study Reports.)

#### Inclusion Criteria

Participants were selected based on their willingness to participate in group meeting times and activities.

#### Exclusion Criteria

Students were excluded for: (1) reporting current NSSI; (2) having lost at least one PE session.

#### Peer Educators

##### Recruitment

Once the school agreed to participate, the program was advertised within the school network–detailing participation requirements and the application process. Eight-grade students had the opportunity to apply to become peer educators. Program advisors delivered an informational meeting. Each aspirant provided a consent form signed by parents. All applicants were invited to participate in a group interview to evaluate their skills and capabilities of serving as peers. Afterwards, a team of selected school staff were asked to rate each student evaluating reliability, leadership, attendance, and ability to carry out the meetings. Eleven peers were selected, but eight (Females = 6, 75%; *M* = 12.71; *SD* = 0.488) confirm their commitment to the program. The peer educators' group was selected in order to be broadly representative of the student body.

##### Peer Educator Training

Once selected, peer educators have received training to acquire the knowledge and skills required to lead PE to younger students. The training was carried out by program advisors (i.e., two psychologists) through a 3 week session, utilizing group activities (i.e., brainstorming, games, role-plays). Also, as peers have to answer questions clearly and correctly, they needed to have an overall knowledge of the subjects, such as puberty and developmental change. Facilitation techniques as active listening and presentation skills were acquired during the training session to strengthen students' capacity as effective peer educators. Communication methods and tools were acquired through discussion and role plays, creating a safe and welcoming environment that allows for an empathetic climate. Program advisors and peers refined and co-created activities within the modules to tailor the PEP to students' needs. Also, involving peers in the decision-making process promotes participation in acquiring skills necessary to implement the program.

During all the training weeks, peer educators could contact program advisors if they needed.

#### Research Participants

##### Recruitment and Assessment

All 6th- and 7th-grade students were invited to participate through school network announcements. An alphanumeric code was assigned to each students who provided assent and parental consent forms. Students were asked to complete a questionnaire booklet in two assessment sessions: baseline (T0) and 1-week post-intervention (T1). The test administration took place during regular school hours, using an online platform (SurveyMonkey), requiring about 40 min to complete. Instructions for each questionnaire were provided, and research assistants were presented at all times.

##### Peer Education Intervention

Peer educators conducted the NSSI-PEP intervention sessions for 5 weeks (2-h sessions per week during school hours). The weekly meetings were centered around the four-core module of the program: (1) self-esteem, (2) body image, (3) pubertal body changes, and (4) emotion regulation. Peers selected the specific weekly topic and activities and conducted the meeting utilizing focus groups, group activities (i.e., brainstorming, games, role-plays), and video. All the sessions culminated with an opportunity for participants to reflect on the experience. Peer educators worked interactively with younger students during the PE sessions, while program advisors supervised the activities and were available if needed. Supplementary Material provides details about the modules' content and activities.

### Outcome Measurements

#### Socio-Demographic Information

Participants completed a socio-demographic sheet asking for information about sex, age, and family composition.

#### Emotion Regulation

The Difficulties in Emotion Regulation Scale (DERS-18; (63)) is a self-report measure assessing emotion regulation difficulties. The DERS-18 constitutes of six subscales–awareness, clarity, non-acceptance, strategies, goals, and impulse–each composed of three items. Participants answered each item on a 5-point Likert scale from “Almost never” (1) to “Almost always” (5). Summing each subscale yields the overall total score, with higher scores indicating higher emotion dysregulation. Victor and Klonsky (64) tested the measure's psychometric properties in five samples and found appropriate internal consistency levels (0.77 to 0.90). In the present study, the Cronbach's alphas ranged from 0.61 to 0.95.

#### Body Image

- The Body Dissatisfaction scale of the Eating Disorder Inventory-3 (EDI-3; (65)) consists of 10 items that assess discontentment with the overall shape and size of the body. Participants rated each item indicated how they agree with statements using a 6-choice format from “Always” (0) to “Never” (4). The Cronbach's alphas ranged from 0.80 to 0.83.

#### Self-Esteem

- The Low Self-Esteem scale of EDI-3 (65) consists of six items assessing feelings of insecurity, inadequacy, ineffectiveness, and lack of personal worth. Higher scores indicate low self-evaluation. In the present study, Cronbach's alpha ranged from 0.89 to 0.90.
- The Personal Alienation scale of EDI-3 (65) consists of seven items measuring emotional emptiness, aloneness, and poor sense of self-understanding. The Cronbach's alphas ranged from 0.79 to 0.83.

#### Puberty

The Maturity Fear scale of EDI-3 (65) consists of eight items assessing the desire to retreat to the security of childhood and return to a pre-pubertal appearance. The Cronbach's alphas ranged from 0.60 to 0.69.

#### NSSI Assessment

At T0, NSSI was assessed using a single-item question: “Have you ever engaged in self-injury without an intent to die?” (yes/no). At T1, an altered version of the item was administered the item “Have you engaged in self-injury without an intent to die since the previous survey?" (yes/no).

#### Program's Adherence, Acceptability, and Feasibility

The NSSI-PEP protocol adherence was assessed through the documentation provided by program advisors. For each meeting, program advisors documented the data, number of participants, module's activities, and the general trend of the meeting. The program's acceptability and feasibility were evaluated through an *ad-hoc* questionnaire assessing program implementation (e.g., “Did the experience meet your expectations?”, “How do you evaluate program's activities,” “How can the program be improved?”, “What would you change?”).

### Analytical Strategy

All the analyses were performed using the IBM Statistical Package for the Social Sciences, Version 26 (66). Quantitative characteristics are reported using means with associated standard deviations, and categorical characteristics are reported using percentages. The internal consistency of each of the subscales was assessed using Cronbach's alpha. Paired sample *t*-test was used to examine the differences from baseline (T0) to post-intervention (T1) across outcome variables. Cohen's *d* was used as the measure of effect size. According to Cohen's rule of thumb (67), the effect size is considered small, medium, or large starting from the value of 0.2, 0.5, and 0.8, respectively. Statistical significance was set at *p* < 0.05; all tests were two-tailed.

Subjects who completed pre-and post-intervention were included in the analyses.

## Results

### Preliminary Analysis

As the NSSI-PEP was a prevention program, individuals were excluded if they reported NSSI. Out of the sample at T0, nine students reported NSSI behavior. They were excluded and were provided with information about available resources in the community, including a university service that provides free-of-charge counseling service.

### Sample Description

At baseline (T0), the sample consisted of 68 students aged between 11 and 13 years old (Females = 26, 38.2%; *M* = 11.59; *SD* = 0.629). At post-intervention (T1) 65 students (Females = 24, 40%; *M* = 11.51; *SD* = 0.562) participated. A total of 58 (Females = 22, 37.3%) 6th- and 7th-grade students completed both baseline and post-intervention assessment. Reasons for exclusion were extracurricular activities in conflict with scheduled modules (*n* = 3), being absent during data collection (T1, *n* = 4), and having lost at least one PE session (*n* = 3). All participants reported being of Italian nationality. More than 80% (*n* = 61) fell into the lower- to upper-middle socioeconomic class.

### Outcomes of NSSI-PEP Intervention

Across the course of the intervention, no students reported new onset of NSSI.

The paired *t*-tests comparing individuals' scores on pre- and post-assessment demonstrated significant changes over the course of intervention. Specifically, students reported statistically significant improvement in emotion regulation abilities [*M*_T0_ = 42.56, *M*_T1_ = 38.53; *t*_(58)_ = 2.126, *p* = 0.038, *d* = 0.27], with less difficulties in acceptance of one's emotions [Non-acceptance subscale; *M*_T0_ = 6.85 *M*_T1_ = 5.43; *t*_(57)_ = 3.006, *p* = 0.004, *d* = 0.40], and greater access to effective emotion regulation strategies [Strategy subscale; *M*_T0_ = 6.56, *M*_T1_ = 4.84; *t*_(57)_ = 4.036, *p* < 0.001, *d* = 0.53]. At post-test, students showed greater self-esteem [*M*_T0_ = 6.32, *M*_T1_ = 2.48; *t*_(58)_ = 4.753, *p* < 0.001, *d* = 0.62], and lower scores (i.e., improvement) on personal alienation [*M*_T0_ = 7.03, *M*_T1_ = 4.21; *t*_(58)_ = 4.141, *p* < 0.001, *d* = 0.55], body dissatisfaction [*M*_T0_ = 9.53, *M*_T1_ = 6.15; *t*_(58)_ = 2.945, *p* = 0.005, *d* = 0.39], and fear maturity [*M*_T0_ = 14.56, *M*_T1_ = 8.98; *t*_(58)_ = 7.055, *p* < 0.001, *d* = 0.93].

Although comparisons on other dimensions of emotion regulation did not reach statistical significance, the mean scores from pre- to post-intervention demonstrated a change in the expected direction (see Table 1 for more details).

**Table 1:** Descriptive statistics and *t*-test results for the study.

**Outcome**	**T0**		**T1**		***t***	***p***	**Cohen'*d***
	***M***	***SD***	***M***	***SD***			
**Emotion regulation**							
Awareness	7.84	3.35	7.84	2.91	−0.008	0.994	<0.01
Clarity	6.35	4.13	6.06	3.35	0.579	0.565	0.07
Goals	8.42	3.85	7.54	3.18	1.835	0.072	0.24
Impulse	7.14	4.16	6.68	3.42	0.868	0.389	0.11
Non-acceptance	6.85	3.98	5.43	2.99	3.006	**0.004**	**0.40**
Strategy	6.56	3.48	4.84	2.32	4.036	**0.000**	**0.53**
Total	42.56	19.76	38.53	13.48	2.126	**0.038**	**0.27**
**Self-esteem**							
Self-esteem	6.32	7.13	2.48	3.47	4.753	**0.001**	**0.62**
Personal alienation	7.03	7.21	4.21	4.65	4.414	**0.001**	**0.55**
**Body image**							
Body dissatisfaction	9.53	9.29	6.15	6.26	2.945	**0.005**	**0.39**
**Puberty**							
Maturity fear	14.56	6.51	8.98	5.40	7.055	**0.000**	**0.93**

*The bold values indicate the constructs measured*.

### Meeting Adherence, Acceptability and Feasibility of NSSI-PEP

Five meetings were conducted between March and May 2021, and attendance ranged from 7 to 22 students to each meeting.

Students reported favorable responses to questions assessing the program's acceptability. All participants indicated that NSSI-PEP fulfills their expectations, deepening all the modules.

More than half of the sample (62.9%) indicated that the meeting's number was adequate and did not interfere with school commitments. About three-quarters of the sample (74.3%) indicated that NSSI-PEP's activities were very comprehensive and pleasant. More than 80% of students would like further to deepen the program's content with peer educators. Overall, the program was evaluated positively (useful: 57.1%, interesting: 65.7%, and educational: 62.9%). No negative aspects/difficulties emerged.

## Discussion

This pilot study examined the effectiveness and feasibility of a school-based prevention program (NSSI-PEP) focusing on vulnerability factors for NSSI, implementing a peer education approach.

The program arises from practical and theoretical assumptions supporting the information-sharing intervention approach's demonstrable ineffectiveness (68). Available programs for NSSI prevention do not demonstrate iatrogenic effects neither reveal any benefits. Alongside such evidence, best practice guidelines for creating preventive interventions suggest that every prevent-effort must be drawn considering contributing factors to the target behavior (68). Notwithstanding, as a nascent field, how to effectively prevent NSSI remains an unanswered question. As such, NSSI-PEP provides a new and innovative model for preventing NSSI among youth, implementing a school-based peer education program targeting areas of vulnerability for the NSSI onset. The program is grounded in the psychoanalytic framework and draws upon the adolescent's developmental tasks' tenets.

Our results suggest that the NSSI-PEP may have promise for being an effective prevention program. Students did not report new onset of NSSI behaviors, and we found that, after the intervention, participants reported a significant decrease in emotion regulation difficulties and reduction on several psychological constructs that have been associated with NSSI. Specifically, students reported a significant decrease in body dissatisfaction. These findings support the effectiveness of treatment approaches targeting positive body image's development: improving body awareness and acceptance may lead to NSSI reduction (69). NSSI has long been conceptualized as a maladaptive strategy to regulate overwhelming emotions *via* the body (70), creating a tangible solution. In this sense, implementing a module addressing abilities to regulate emotions is beneficial as our results support a significant change in emotion regulation abilities: students reported increased capability to accept emotions and access to more effective emotion regulation strategies. Also, from pre- to post-intervention, students reported changes in other dimensions of emotion regulation (i.e., awareness, clarity, goals, and impulse), even though they did not reach statistical significance.

Also, the significant change in the level of self-esteem from pre- to post-intervention corroborates the protective role of such a factor. Higher self-esteem was indeed found as an intrapersonal factor predicting NSSI cessation 12 months later (71). Results evidenced lower scores on personal alienation, suggesting a reduction in emotional emptiness and feelings of being separate from others. These findings reflect a positive change in self-evaluation and a greater sense of closeness to others and self-understanding, reflecting crucial factors in identity formation during adolescence (72).

Lastly, after the intervention, students reported a significant change–with a large effect size–in maturity fear, suggesting a reduction in the fears, turmoil, conflicts, and developmental expectations related to the several puberty's changes for which adolescents feel ill-prepared.

During the sensitive pubertal phase, adolescents go through less to more complex changes. According to Freud (73), puberty requires adjustments and new arrangements linked to the physical, sexual maturity and the potential to procreate. Past psychological development needs to be integrated into a new sexual body throughout adolescence, reorganizing wishes and fantasies. The sexual body, during adolescence, becomes the crossroad of a surplus of excitement (29). In this sense, the final sexual organization–integrating the infant body with the sexual body –represents the adolescent's primary developmental function. Integrating the sexual body requires a complex work of symbolic reorganization that adolescents are called to face to accomplish the developmental function. Severe interferences in such a process could result in a developmental breakdown (74). The inability to deal with the symbolization process (64) may lead to a fracture in the identity continuity of the adolescent.

As a result, being unable to integrate the physically mature body image into the representation of oneself is often expressed by moving the painful and frightening fantasies and emotions into the body that is emotionally disinvested: adolescents experience a kind of war which also contains a hatred of oneself and one's own body, as an enemy and source of worthlessness (75). Thoughts and feelings coming from the body are overwhelming, and, in the face of these demands, the adolescent may feel unprepared and helpless, finding a solution “through the body,” possibly resulting in self-destructive behaviors (29), such as NSSI. Moreover, besides being attacked, the body may be mortified in its appetites, deprived of passions and desires, as in eating disorders (76).

A distorted relationship with the body, arising from feelings of worthlessness and inadequacy, impairs emotion regulation processes. Indeed, a growing body of research supports the assumption that body disregard is strongly associated with the occurrence of self-destructive behaviors, such as NSSI and eating disorders, in the context of emotion dysregulation (44).

Although we did not detect significant results on other risk factors, such as dimensions of emotion regulation, participants reported changes in the expected directions.

Thus, the preliminary results from this study are encouraging and support the notion that school-based program implementing an interactive approach (peer education) can be potentially effective in preventing NSSI among youth.

Whilst peer educators, as someone “in the know,” effectively delivered PE intervention, there are significant challenges we have encountered in implementing the training. First, three selected peers decided to give up the program, as they perceived the PE needs exceeding their capability. Second, striking balance between empowerment and training took time, investment, and ongoing support. Lastly, although the online delivery of the training was cost-effective and easily accessible, the virtual environment limited the students-to-student and student-to-adult interactions and made it more difficult to develop connections and work with study's constructs, such as body image. In order to address these shortcomings, the PE should be in presence to foster and maintenance empathetic relationships with participants and maximize the effectiveness of the training.

Cumulatively, implementing a preventive strategy addressing the underlying mechanisms of NSSI has a leading role in transforming the traditional preventive model. In this sense, a remarkable starting point concerns the use of a peer education approach: the NSSI-PEP is grounded in the belief that young people gain more from an experience when they are actively involved. Thus, the PE approach provides the opportunities to belong and experience a supportive relationship (for both peers and target group) to develop and practice new skills and improve self-efficacy.

### Strengths and Limitations

The program presents many strengths. First of all, it has a robust theoretical background that meets the empirical findings collected within the NSSI literature. Second, it addresses preadolescence, the age range with a greater risk of self-injurious behaviors and aims to prevent these behaviors using an innovative methodology. Findings suggest an urge for early prevention as youngsters engaging in NSSI usually do not seek help (10), with just 17% of adolescents receiving professional support for self-injury (77). Also, peers have been shown to play a crucial role in youth psychosocial development since they can affect each other's feelings, habits and behaviors and exhibited a significant impact on NSSI engagement (78). Thus, their inclusion in NSSI prevention initiatives is considered to be of crucial importance (16). For these reasons, peer education appears to be a potentially cost-effective instrument to deliver a preventative intervention by sharing knowledge through personal experience and creating a trustful and horizontal relationship.

Another strength is focusing on NSSI risk factors to reduce youngsters' chances of engaging in self-injurious behaviors. However, even though researchers suggest addressing vulnerability factors within preventive interventions (18), to our knowledge, no current prevention study addressed such NSSI variables.

However, the study also has several challenges and limitations. First, participation in the intervention is quite time-consuming for the school. Second, to test the generalizability of the intervention, the program will need to be extended to a greater diversity of school implementing longer and more frequent follow-ups. Third, we were not able to enroll a second school to act as a control group. Thus, further studies need to implement a randomized control trial design including a control group. Fourth, we have only two time-points limiting the conclusions about the data. A refinement of PEP is needed to increment assessment point and involve a larger number of participants. Fifth, samples' selection (peer educators and students) is prone to volunteer bias and may limit the generalizability of the results.

Despite these limitations, our pilot study suggests that NSSI-PEP represents a promising prevention program to integrate into school systems to enhance protective factors and resilience among youth. Also, such encouraging results shed new light on the relevance of implementing a program based on the underlying mechanisms that drive the complex processes of pubertal development. Clinicians and practitioners would be provided with a novel approach not only to fighting NSSI but also to developing positive, sustainable health behaviors in youth and promote psychological well-being and a healthy lifestyle over time, producing sizable and sustained benefits to society.

## Data Availability Statement

The raw data supporting the conclusions of this article are available from the corresponding author, SC, upon reasonable request.

## Ethics Statement

The studies involving human participants were reviewed and approved by Department of Psychology, University of Campania Luigi Vanvitelli. Written informed consent to participate in this study was provided by the participants' legal guardian/next of kin.

## Author Contributions

SC and PC designed the study. SC wrote the protocol. AC and LB conducted the study's activities. CA reviewed the literature and provided summaries of previous research studies. AC conducted the statistical analysis. SC and AC wrote the first draft of the manuscript. All authors contributed to and have approved the final manuscript.

## Funding

This research was supported by V:ALERE 2020 (VAnviteLli pEr la RicErca)-University of Campania Luigi Vanvitelli [Legislative Decree No. 475 of 9 July 2020]. The funding source had no role in the design and conduct of the study, collection, management, analysis, interpretation of the data, preparation, review or approval of the manuscript, and decision to submit the manuscript for publication.

## Conflict of Interest

The authors declare that the research was conducted in the absence of any commercial or financial relationships that could be construed as a potential conflict of interest.

## Publisher's Note

All claims expressed in this article are solely those of the authors and do not necessarily represent those of their affiliated organizations, or those of the publisher, the editors and the reviewers. Any product that may be evaluated in this article, or claim that may be made by its manufacturer, is not guaranteed or endorsed by the publisher.
