# Supplementary material for: Non-Suicidal Self-Injury: A School-Based Peer Education Program for Adolescents During COVID-19 Pandemic
Source: Front Psychiatry. 2022 Jan 13;12:737544. doi: 10.3389/fpsyt.2021.737544 (PMC8793799; doi:10.3389/fpsyt.2021.737544)
Supplement: Supplementary file 1 [file Table_1.DOCX]

Supplementary Material

**Table**. Description of modules’ contents.

| **Module Topic** | **Description** |
| --- | --- |
| **Self-esteem** | The Self-Esteem Module aims to help students building a healthy sense of self-esteem.  Example of activities includes:  - Focus on strengths and values  - Identifying and challenging personal values |
| **Body Image** | The Body Image module is designed to explore all the dimensions of body image (e.g., satisfaction/dissatisfaction, appearance, emotional investment) in order to help students to develop a positive body image.  Example of activities includes:  - Group plays (e.g., Taboo, crosstabs) |
| **Pubertal Body Change** | The Pubertal Change module is designed to help students addressing all the physical (also emotional and social) changes that occur during puberty (e.g., menstruation, secondary sexual characteristics).  Example of activities includes:  - Write “a letter to my body” |
| **Emotion Regulation** | The Emotion regulation module addresses all the emotion’s regulation skills: recognition, understanding, and managing emotions. Specifically, all the activities aim to help students to recognize emotional states and learn how to manage, express, and cope – accessing functional strategies – with such emotions, especially negative ones.  Example of activities included:  - Emotional vignettes/situation |
